# TRP Channels as Interior Designers: Remodeling the Endolysosomal Compartment in Natural Killer Cells

**DOI:** 10.3389/fimmu.2020.00753

**Published:** 2020-04-28

**Authors:** Dennis Clement, Jodie P. Goodridge, Christian Grimm, Sandip Patel, Karl-Johan Malmberg

**Affiliations:** ^1^The KG Jebsen Center for Cancer Immunotherapy, Institute of Clinical Medicine, University of Oslo, Oslo, Norway; ^2^Department of Cancer Immunology, Oslo University Hospital, Institute for Cancer Research, Oslo, Norway; ^3^Fate Therapeutics Inc., San Diego, CA, United States; ^4^Faculty of Medicine, Walther Straub Institute of Pharmacology and Toxicology, Ludwig-Maximilians-Universität, Munich, Germany; ^5^Department of Cell and Developmental Biology, University College London, London, United Kingdom; ^6^Department of Medicine Huddinge, Center for Infectious Medicine, Karolinska Institutet, Stockholm, Sweden

**Keywords:** NK cell, TRP cation channel, secretory lysosomes, cytotoxic lymphocytes, mucolipin, calcium signaling

## Abstract

Cytotoxic lymphocytes, including natural killer (NK) cells and T cells are distinguished by their ability to eliminate target cells through release of secretory lysosomes. Conventional lysosomes and secretory lysosomes are part of the pleomorphic endolysosomal system and characterized by its highly dynamic nature. Several calcium-permeable TRP calcium channels play an essential role in endolysosomal calcium signaling to ensure proper function of these organelles. In NK cells, the expression of self MHC-specific inhibitory receptors dynamically tunes their secretory potential in a non-transcriptional, calcium-dependent manner. New insights suggest that TRPML1-mediated lysosomal calcium fluxes are tightly interconnected to NK cell functionality through modulation of granzyme B and perforin content of the secretory lysosome. Lysosomal TRP channels show a subset-specific expression pattern during NK differentiation, which is paralleled with gradually increased loading of effector molecules in secretory lysosomes. Methodological advances, including organellar patch-clamping, specific pharmacological modulators, and genetically-encoded calcium indicators open up new possibilities to investigate how TRP channels influence communication between intracellular organelles in immune cells. This review discusses our current understanding of lysosome biogenesis in NK cells with an emphasis on the TRP mucolipin family and the implications for NK cell functionality and cancer immunotherapy.

## The Secretory Lysosome at The Heart of Intracellular Calcium Signaling in NK Cells

Malignant or virally-infected cells can be eradicated by T cells, due to surface presentation of peptide-loaded major histocompatibility complex (MHC) class I molecules (1, 2). However, some cells escape T cell recognition through down-regulated expression of MHC class I molecules, due to specific genetic alterations, cellular stress, or intracellular retention by viral proteins. This potential loophole of the body's defense is guarded by natural killer (NK) cells that express MHC binding inhibitory receptors. Therefore, in contrast to T cells, it is the loss of MHC expression that can elicit a cytotoxic response by NK cells (3). NK cells also recognize discontinuity such as expression of stress ligands commonly displayed on virally infected cells through a broad array of activating receptors (4). Hence, NK cell responses are counterbalanced by combinations of various inhibitory and activating signals (5, 6). The net outcome of these signaling cascades that drive these responses is reflected in a shift of the cytoplasmic calcium homeostasis. Unopposed triggering of NK cells by ligands for activating receptors evokes strong calcium signals in the cytoplasm of NK cells (7–9).

At the interface between the effector and target cell lysosome-related organelles polarize and fuse with the plasma membrane in a highly-directed, and calcium-dependent manner (2, 10, 11). The release of lysosome-related organelles (or lytic granules), hereafter referred to as secretory lysosomes, represents one of the classic killing mechanisms of NK and T cells and is mostly driven by ER-derived calcium stores (11, 12). Successful release of cytotoxic molecules marks a crucial step in the destruction of the target cell (13). Additional evidence suggests that a single NK cell degranulation event is sufficient to trigger target cell apoptosis, as elucidated by elegant live cell imaging (14).

However, the underlying global calcium signals are far more complex and require not just the ER-derived calcium, but also involve calcium signals from the acidic calcium stores (15). The importance of calcium signals derived from the acidic, endolysosomal calcium stores, as a basis for communication between organelles and coordination of metabolism and transport processes has become increasingly appreciated in the context of disease and immune cell function (16–19). A multitude of metabolites can be exchanged via physical inter-organelle contact sites, which, in turn, influences calcium channel activity, and can help to coordinate calcium homeostasis, in order to establish proper immune cell responses. In cytotoxic lymphocytes, changes in the lysosomal signaling capacity directly correlates with immune cell effector responses (20–25). Therefore, lysosomal calcium channels emerge as novel targets for genetic and pharmacological interventions aiming to boost immune effector cell function in cancer immunotherapy.

This review focus on the role of transient receptor potential (TRP) calcium channels in the remodeling of the endolysosomal compartment in NK cells and in orchestrating organelle communication in cytotoxic lymphocytes. It is important to point out that the lysosomal calcium channel repertoire is highly diverse. In addition to TRPs, which are the focus of the current review, they also comprise two pore channels (TPCs) and the ATP-gated P2X channel family (16). In T cells the TPC calcium channel, TPC2, has been detected on secretory lysosomes and TPC2-activation stimulates the calcium-dependent exocytosis of the these organelles (26). TPC biology and their role in the endolysosomal system are reviewed elsewhere (27, 28).

## The Lysosomal Compartment in Cytotoxic Lymphocytes

Early studies have revealed important morphological features and the functional principle of secretory lysosomes in cytotoxic immune cells. Electron microscopy enabled examination of these organelles in great detail, and as the name implies, morphologically they resemble conventional lysosomes in many aspects. Yet, there is a large degree of heterogeneity within the pool of lysosomes, as discussed later in this section (29, 30). Moreover, both conventional lysosomes and secretory lysosomes share vesicular delivery pathways from the Golgi and represent the terminal part of the endosomal pathway (30–32). This model is based on the immuno-EM detection of lytic effector molecules, an electron-dense core and acid hydrolases in the same compartment, which supports a common identity. Hence, secretory lysosomes in T and NK cells are lysosomes with a luminal secretory unit, the dense core, which contains the effector molecules and is releasable during degranulation (33–35). The electron dense core of secretory lysosomes is mainly composed of serglycin, an anionic chondroitin-sulfate coated proteoglycan, regarded to function as safe storage site for cationic granzymes and perforin (36, 37). Lysosomes are a heterogenous group of organelles distinguished on the basis of their luminal pH, morphology, degradative capacity, and subcellular positioning (38). Furthermore, the secretory lysosomes of NK cells have been described as a multi-facetted pool of organelles and are commonly divided into three classes (33). Type I granules appear to have an electron-dense core with relatively little cortex. In comparison, type II granules are distinguished with varying morphology and inclusion of vesicles and membrane whorls. First, the intermediate type of granules combines both phenotypes and can appear with multiple dense-core structures and vesicular, multilamellar cortex. Second, it has been speculated that type I granules might represent a more mature stage, whereas, intermediate and type II might resemble immature stages in the cycle of granule biogenesis (39). The interpretation of this model must be treated with some caution, since NK cell specific data has mostly been obtained from investigations on two-dimensional images of rat NK cell lines or on PBMC fractions. In future studies the implementation of correlative-light electron microscopy and three-dimensional EM-tomography of primary well-defined NK cell subsets might provide additional insights on the heterogeneity of dense core granules.

## Remodeling of Secretory Lysosomes in NK Cells

Lysosomes in NK and T cells have dual functionality. These acidic organelles harbor both the enzymatic activity of a conventional lysosome and cytotoxic molecules, most importantly granzyme B and perforin, used for killing target cells by apoptosis. Target cell recognition unleashes a complex signaling cascade in cytotoxic lymphocytes, which culminates in the formation of an immunological synapse (IS). Most importantly, PLCγ-generated calcium signals derived from IP_3_-receptors (Inositol triphosphate receptor) at the Endoplasmic reticulum (ER) are one of the key downstream signals that are paramount in orchestrating the directed NK cell degranulation at the IS (40–42). Cytotoxic lymphocytes recruit further proteins to the lysosomal surface, which primes these organelles for exocytosis. In NK cells, among these accessory proteins are the small GTPase Rab27a and its effector Munc13-4, which interact with the SNARE proteins syntaxin11 and VAMP7 on the plasma membrane (43–46). The Rab27a-dependent mode of secretion discriminates secretory lysosomes from conventional lysosomes (45). These peripheral proteins on the cytoplasmic side of the secretory lysosome are needed to initiate transportation toward the IS, followed by a docking step and fusion with the plasma membrane (10, 11, 47, 48). By contrast, specialized secretory cells, such as insulin secreting β cells of the pancreas, contain conventional lysosomes for digestion of endocytosed cargo, alongside a designated pool of vesicles for regulated release of their secretory products.

Naïve T cells undergo an initial priming phase that requires stimulation of the TCR in order to initiate secretory lysosome formation along with accumulation of effector molecules (49). In contrast to naïve CD8^+^ T cells, NK cells generate granzyme B and perforin containing secretory lysosomes in response to cytokines alone, without the need for specific ligation of an activating receptor (30, 33, 50). The cytotoxic potential of NK cells develops gradually during differentiation and is reflected in the content of effector molecules and the accumulation of large secretory lysosomes (37, 51, 52). In addition to this gradual acquisition of effector function, NK cells tune their functional potential against self MHC in a non-transcriptional process termed education (53–57). Inhibitory interactions with self-MHC translate into a predictable, quantitative relationship between self-recognition and effector potential. Paradoxically, a lack of constitutive inhibitory self-interaction is associated with hyporesponsiveness of NK cell subsets to various stimuli, while the presence of self-interactions is associated with gain in functionality. Although there are several models that encompass cellular events that are critical for NK cell education, the mechanism behind the functional calibration against MHC has remained unclear. Recent work from our laboratory established a direct link between inhibitory signaling and the size of the secretory lysosomes in NK cells (25). We found that educated NK cells contain more granzyme B stored in a unique pool of dense core secretory lysosomes. Furthermore, as we shall discuss in more detail below, pharmacological and genetic interference pointed to a previously unknown upstream role for Transient Receptor Potential Mucolipin-1 (TRPML1) activity in the remodeling of secretory lysosomes in NK cells. Altogether, new mechanistic insights were gained from studying NK cell education, which demonstrated that tonic inhibitory receptor input from the plasma membrane can affect critical processes in the endolysosomal system, with sustained impact on the calcium signaling capacity from the acidic compartment.

## Lysosomes at The Center Stage of Metabolic Regulation

**Figure 1:**
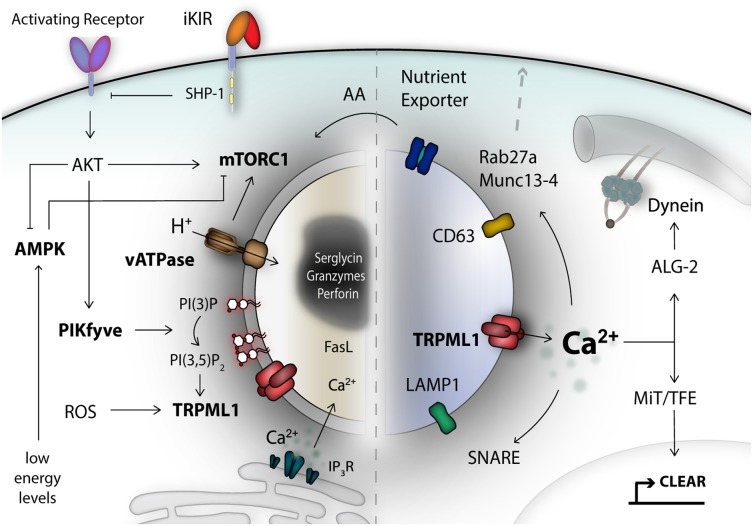
The lysosomal compartment is an important signaling hub and integrates a diverse range of signals. Secretory lysosomes are dual-functional organelles consisting of a lysosomal limiting membrane and a proteoglycan electron-dense core as safe storage unit for effector molecules like granzymes and perforin. Many different signals form the cell surface, or from the inside, converge at the limiting lysosomal membrane and can be detected by specialized metabolic-, energetic-, stress-, pH-, and lipid-moiety-sensor proteins. For NK cells, one of the central metabolic sensors is called mTOR complex 1, which can detect amino acids (AA) and growth factor signals. AMP-activated kinase (AMPK) reacts to stress signals, such as reactive-oxygen species (ROS) and can trigger autophagy induction to recover nutrients. A remarkable class of signal integrators, is the transient receptor potential (TRP) channel family, most importantly TRPML1, localized on the lysosomal membrane. TRP channels can integrate signals of diverse nature, translated into calcium signals. TRPML1 calcium signals control lysosomal trafficking membrane dynamics and TFEB-dependent activation of the CLEAR gene network. A network of genes associated with lysosomal biogenesis and autophagy, and commonly regulated by transcription factors of the MiT/TFE family. Lysosomal calcium signals and lipid membrane composition, as well as integral lysosomal surface proteins are essential for the recruitment of e.g., motor proteins, the small Rab27a GTPase, Munc 13-4, and SNARE proteins as mediators of plasma membrane fusion. Altogether, these are critical components for orchestrating exocytosis of secretory lysosomes in NK cells. iKIR, inhibitory killer immunoglobulin-like receptors.

The highly complex nature of the lysosomal compartment and how it shapes cellular calcium signaling was initially largely overlooked and lysosomes were simply regarded as terminal unit for degradation of endocytosed material. The original concept is reflected in its name “*lysis-* and -*some*" derived from the Greek for “digestive body” (58). Since then, our view on lysosomes has changed dramatically, from a waste disposal site to a multifunctional signaling hub, indispensable for cellular calcium signaling and killing ability of cytotoxic lymphocytes, and at the center stage of metabolic control (Figure 1). Secretory lysosomes can be seen as a two-component organelle combining the luminal constituents and outer limiting membrane of a conventional lysosome with an electron dense-core, harboring toxic effector molecules (33–35). Intriguingly, there are mechanisms in place to maintain lysosomal integrity, due to their cytotoxic load (59). Irrevocably damaged lysosomes will be subject to lysophagy (60, 61).

Secretory lysosomes are organelles with dual functionality and they have a similar biogenesis as conventional lysosomes. Lysosomal biogenesis is a highly dynamic process, which incorporates a myriad of different cellular signaling pathways and metabolic conditions, which are surveyed by intracellular metabolic sensor proteins. One of the key metabolic sensors is called mechanistic target of Rapamycin (mTOR) (62, 63). NK cell maturation and responsiveness to cytokine-mediated activation and proliferation is critically dependent on mTOR (64, 65). The active mTOR kinase complex is recruited to the lysosomal surface in order to sense nutrient and growth factor input (63, 66). During starvation, a lack of nutrients and low energy levels are detected by AMPK. A complex signaling cascade, encompassing AMPK and mTORC1 and lysosomal pH changes, promote lysosomal biogenesis and autophagy in a coordinated fashion, allowing recovery of nutrients (67). The reformation of lysosomes after termination of autophagy has been linked to reactivation of mTORC1 (68). Moreover, this process also integrates transcription factor cues, such as TFEB, which regulates expression of a network of genes for lysosomal biogenesis and autophagy, termed the CLEAR network (69). Nutrient levels like cholesterol can also be sensed and regulate lysosome motility via TRPML1-derived calcium signals and subsequent ALG-2-dependent dynein engagement (70). Damaged mitochondria can prompt TRPML1 activation by reactive-oxygen species (ROS) and orchestrate lysosomal adaptation to clear damaged mitochondria via autophagy, known as mitophagy (71). Altogether, this illustrates a cross-talk of fundamental metabolic-, pathogenic- and stress-signals at the lysosome, which are jointly integrated and aim to establish a stable lysosomal number matching the cellular needs (72, 73).

## The Lysosome as a Dynamic Functional Unit in The Endolysosomal System

**Figure 2:**
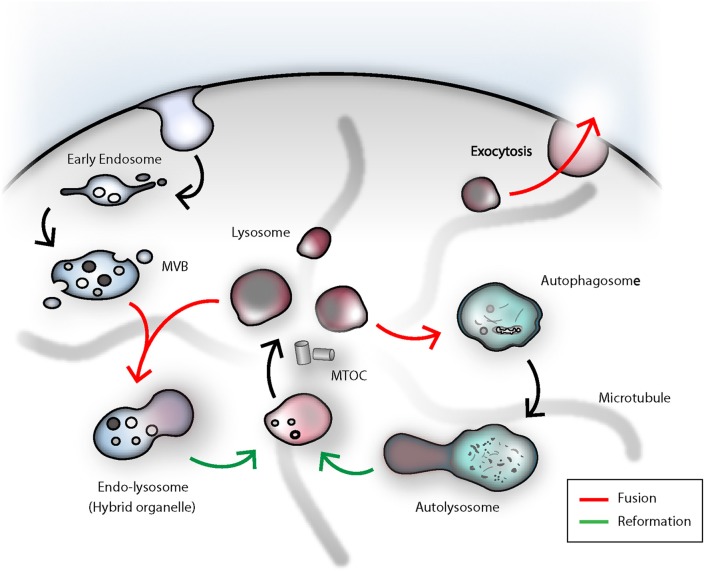
The dense-core secretory lysosomes are the terminal storage unit for acidic hydrolases and effector molecules in NK cells. The lysosome as such is a highly dynamic compartment and can engage in several fusion events in order to deliver its degradative capacity for endocytosis, autophagy or killing of target cells by NK cells. Termination of signal transduction is an important task for the orchestration of NK cell effector functions. Early endosomal cargo can be propagated by gradual-maturation of an early endosome to a late endosome, which finally can fuse with a lysosome for degradation by acidic hydrolases. As a result, an endolysosomal hybrid organelle is formed. Lysosomes can be reformed through tubulation from the latter compartment and leads to formation of a proto-lysosome, which undergoes further maturation and content condensation. In analogy to that, lysosomes can also engage in autophagy. In immune cells, autophagy fulfills the important task of eradicating worn-out mitochondria. Damaged organelles or proteins can be entrapped in autophagosomes, ultimately fusing with lysosomes. Upon termination of autophagy, again, through tubulation and membrane extrusions, consumed proto-lysosomes are regenerated and mature to lysosomes. During target cell killing, NK cells go through step-wise receptor cross-linking, granule polarization and eventually lysosome-related organelle fusion with the plasma membrane at the immunological synapse. During this process, the effector molecules and the electron-dense core can be ejected and the lysosomal membrane will be recovered through endocytosis. MVB, multi-vesicular body; MTOC, microtubule-organizing center.

The classical pathway of endocytic cargo trafficking from the early endosome to the lysosome is based on a gradual maturation model. First, endocytosed material is collected in the early endosome, which then is subject of recycling back to the plasma membrane or undergoes ESCRT-complex-mediated sorting of ubiquitinylated proteins into intralumenal vesicles (ILV) (74, 75). During the transition from early to late endosome, endosomal proteins are retracted and newly synthesized lysosomal hydrolases, and in the case of secretory lysosomes, also effector molecules, are received from the Golgi complex. Due to homotypic fusions between LEs, an increasing size and a higher density of ILVs dominate the appearance of late endosomes or so-called multi-vesicular bodies (MVB). Late endosomal acidification and increase of the luminal calcium concentration through intensified ER-contact sites, is another prerequisite prior to fusion with a lysosome (30, 76–78). In a final step a temporary hybrid organelle called endolysosome is generated via transient kiss-and-run or full-fusion events between late endosomes and lysosomes. Concurrently, this marks the final step in the degradative pathway and commences the lysosomal reformation from the endolysosome (67, 79–81). Moreover, the lysosomal number and size, as well as positioning within the cells are critical parameters (70, 73, 82). The constant adaptation of the lysosomal compartment, regulated by the cellular demands, is imperative for proper cellular function. Since only membranes in close proximity can undergo fusion, lysosomal motility is required for degradation of damaged organelles and endocytosed cargo. Lysosomal trafficking is coordinated by kinesin and dynein proteins, which are recruited to the peripheral lysosomal membrane in a calcium- or nutrient-dependent manner (70, 83, 84). The very same proteins involved in tethering motor proteins to lysosomes are indispensable for granule convergence to the MTOC (Microtubule-organizing center) in NK cells (85). From this perspective, lysosomes can be regarded as dynamic functional unit, which can be transported on microtubule tracks to the place of action (84). Mechanistically, however, the process of lysosomal fusion and fission is not well-defined. Hitherto, several mechanisms have been postulated and will most likely be context-dependent, or apply only to a fraction of lysosomes within a given cell (86). In fact, the number of freely available lysosomes in a cell is depleted, each time, a lysosome undergoes homotypic or heterotypic fusion with late endosomes or autophagosomes, or else is secreted (Figure 2). The actual biogenesis and size modulation of new lysosomes from existing lysosomes or transient hybrid organelles has been understood as a reformation process. Lysosomal reformation, by fission, from an existing late endosomal- or autophagosomal-pool can take place through tubulation and subsequent calcium-dependent scission of these tubular membrane extrusions, which bridge the donor endolysosome and the newly formed protolysosomes (80, 87, 88).

Above, we have discussed the regulation and function of conventional lysosomes as well as some specific features of secretory lysosomes found predominantly in immune effector cells such as T and NK cells. The lysosomal compartment in cytotoxic lymphocytes is dominated by secretory lysosomes (35). It is therefore reasonable to assume that secretory lysosomes carry out all the classic lysosomal functions, such as metabolic control, degradation of endocytosed cargo and autophagy, on top of their unique role in the killing of target cells. Whereas, T cells have to undergo antigen-driven activation in order to form dense-core secretory lysosomes (49), all mature NK cells contain preformed dense-core secretory lysosomes with high levels of perforin and granzyme B (50, 51). Notably, CD56^bright^ NK cells, which are less differentiated NK cells, drastically upregulate the granular content upon short term exposure to IL-15 (89). It is difficult to discriminate conventional lysosomes from secretory lysosomes solely based on morphological features, since the electron-dense core of secretory lysosomes can have a wide variety of shapes (33, 35). Instead, secretory lysosomes can be discriminated from conventional lysosomes based on the unique protein machinery involved in the tightly regulated mode of secretion (35). The main evidence for this notion can be deduced from genetic diseases that only impair the secretion of secretory lysosomes, but not the secretion of conventional lysosomes. Mutation of the RAB27A gene, leads to immunodeficiencies and hypopigmentation. The manifested disease phenotype is known as Griscellis syndrome, which only strikes and compromises secretion of secretory lysosomes in immune cells and melanocytes (90). Similarly, much could be learned about the specific function of secretory lysosomes from studies in patients with Chediak-Higashi Syndrome (CHS). The phenotype of CHS includes defects in skin pigmentation and immunodeficiencies due to malfunction of the release of secretory lysosomes with no impact on conventional lysosome function (35, 91, 92). Altogether, both disease phenotypes indicate a common theme of secretion that involves an exclusive secretory apparatus, which is used by secretory lysosomes, as found in cytotoxic lymphocytes and melanocytes (10). From an evolutionary viewpoint, the development of the secretory lysosomes, probably originated from conventional lysosomes, and give cytotoxic lymphocytes and other cell types the ability to control the surface mobilization of lysosomal membrane proteins and soluble, luminal effector molecules in a spatiotemporal fashion (35, 93).

## Calcium Signals Fine Tune Functional Responses in Immune Effector Cells

The ER is the most-studied and well-established principal calcium source in NK cells. Stimulation of NK cells triggers ER-derived calcium efflux (40–42) Ultimately, this results in depletion of ER calcium stores, which subsequently triggers a well characterized process, called store-operated calcium entry (SOCE), and replenishes ER calcium levels by tapping into extracellular sources of calcium via ER-plasma membrane contact sites (94, 95). Defects in this process, strongly affect NK cell degranulation and cytokine production (96). The contribution of individual calcium sources and the interplay between the ER calcium stores and the acidic, lysosomal compartment, as well as various other intracellular calcium sources is covered in several excellent reviews (97–99). Increasing evidence suggests that the acidic compartment of the endolysosomal system plays an important role in intracellular calcium signaling. The latter compartment contains high amounts of luminal calcium, quantified at around 0.5 mM, which corresponds to calcium concentrations similar to the ER at steady-state conditions (100–102). However, when compared with the low cytosolic calcium concentration of approximately 100 nM, it becomes apparent that signals derived from endolysosomal calcium flux can influence cytosolic events. It is not surprising that permeabilization of lysosomes strongly raises cytosolic calcium levels and can provoke secondary ER-derived calcium flux likely at membrane contact sites between the two organelles (15, 97). Conversely, the refilling of lysosomal calcium stores may also have important implications for signaling. ER-derived calcium signals can be sequestered by lysosomes and, consequently, alter cytosolic calcium signals (103, 104). Indeed, there is evidence that the ER is the principal calcium source for filling lysosomes (105). However, the molecular mechanisms underlying lysosome calcium uptake are unclear. Most evidence suggests that lysosomal calcium uptake occurs through Ca^2+^-H^+^ exchangers but these genes have yet to be identified in humans (106). Alternative routes involve the Parkinson's related protein, ATP13A2 (107). Many endolysosomal processes are coordinated by calcium fluxes (108, 109). These encompass lysosomal positioning, reformation and membrane fission or fusion events (67, 69, 70, 88). Calcium channels of the TRP family are one of the most versatile group of ion channels in terms of integration of disparate signals, including phosphatidylinositol phosphate levels and pH-changes. The lysosome fulfills fundamental functions for the immune system, including antigen processing for MHC class II presentation, the release of cytotoxic granules and influences migration of immune cells (110). Many of these cytosolic events are centered around the calcium-permeable TRP channel family (111).

Lysosomal calcium signals are essential for proper target cell killing by NK cells (20, 25). Alterations of lysosomal calcium signaling, as seen during lysosomal storage disorders, mitigate proper execution of NK cell effector functions, due to lysosomal impairment (20, 101). In addition, eradication of ROS by mitophagy, a process dependent on lysosomes, contributes to survival of virus-specific NK cells during NK cell memory formation (112). The number and size of lysosomes may vary drastically between the resting state of immune cells in the blood stream and during tumor-challenge within the debilitating, nutrient-depleted tumor microenvironment (113). In the next section, we will elaborate on the molecular pathways by which the lysosome-derived calcium signals affect the outcome of immune effector cell responses.

## Function of Distinct TRP Channels in Cytotoxic Lymphocytes

The transient receptor potential (TRP) channels comprises a functionally versatile superfamily of 28 disparate members and are classified in six subfamilies. All TRP channels possess six putative transmembrane domains and assemble as tetrameric complexes with cation-permeable pores (114). The channel activity underlies a wide variety of stimuli and can integrate several signals at once. The TRP superfamily shows diverse ion selectivity, and hence, are not just exclusive calcium channels (115, 116). TRP cation-channels are known to be broadly expressed among immune cells (111). Yet, the repertoire of TRP channels in immune cells, especially NK cells, remains poorly characterized. TRPML1, TRPML2, and TRPM2 in particular have been implicated in the regulation of innate immunity, and NK cell effector functions.

Cation-channels are the door opener of immune reactions, since calcium-mobilization is crucial for proper lymphocyte activation and function, such as degranulation, cytokine-production, and proliferation (117). TRP channels have the capability to directly alter the intracellular calcium levels and consequently modulate fundamental signaling processes that orchestrate, among other things, differentiation, migration, and cytotoxic effector responses of dendritic cells, neutrophils, macrophages, NK cells, CD8^+^ T cells and mast cells (21, 118–120). There are several excellent and comprehensive reviews describing the clinical picture of TRP channelopathies and the impact on the immune system (111, 116, 117, 121–124). TRP channels moved into the spotlight of pharmaceutical research due to their pathophysiological role in the sensory nervous system with implications on chronic pain disorders (121). Congenital mutations in TRP channels can lead to severe disease phenotypes and can affect disparate tissues (116). This facet is also reflected in the name of two TRP subfamilies, the -mucolipin (TRPML) and -polycystic subfamily (TRPP), which were both named according to the related human diseases: mucolipidosis and polycystic kidney disease (121). Derailed TRP channel sensitization can contribute to the secretion of neuropeptides, such as CGRP and substance P, which can trigger neurogenic inflammatory responses. As a result, these neuropeptides can locally modulate vascular permeability, leukocyte migration and immune cell activation (125). The underlying inflammatory processes are complex and have been associated with miscellaneous diseases, in particular, inflammatory bowel disease, asthma, arthritis, experimental autoimmune encephalomyelitis and anaphylaxis [reviewed in (111)].

The detailed mapping and functional characterization of TRP channels in primary human immune cells is still incomplete. New evidence based on gene-deletion studies helped to uncover the role of TRP channels in the immune system and in inflammatory conditions. For example, the targeted-deletion of TRPM7 in T cells in a mouse model, leads to reduced peripheral T cell numbers due to impaired T cell selection and development in the thymus (126). TRPM7-mutant T cells showed altered migratory behavior and affected mice suffered from pulmonary inflammation, most likely based on defects in Fas-dependent T cell apoptosis (123). These findings underline the importance of TRPM7 in lymphocytes and were extended in DT40 chicken B cells, where genetic deletion of TRPM7 leads to increased cell death and compromised B cell proliferation (127). Further mouse studies have shown alterations in innate immune cell function, such as mast cell responses toward IgE and monocyte-derived chemokine release, by genetic manipulation of TRPM channels (117). Taken together, TRP channels can influence the response of individual immune cells and contribute to the homeostasis of inflammation in health and disease. Mutations or impairment of single TRP channels can lead to serious congenital diseases and have also been associated with the pathophysiologic course of various inflammatory diseases (111, 121). All these properties make TRP proteins attractive targets for immunomodulatory drugs and genetic manipulations.

Exposure of NK cells to activating stimuli triggers complex signal transduction and provokes effector responses (6). A diverse range of input signals converge at the level of the lysosome, which subsequently orchestrates appropriate cellular responses (72, 73, 128). We propose that the nature of these upstream signals, in concert with the highly dynamic nature of the lysosome, are imprinted through remodeling of the lysosomal membrane and the components making up the dense-core. Therefore, the composition of the lysosomal compartment has profound impact on immune cell responses that extends beyond the release of the effector molecules stored in the granules (25). This notion is supported by the fact that lysosomal constituents provide a critical molecular wiring, translating upstream signaling into appropriate downstream functionality, with TRP channels acting as key signal integrators (116). In essence, the lysosomal compartment is a tunable signaling hub and can be remodeled by external stimuli or rational manipulation.

### TRPML1

**Figure 3:**
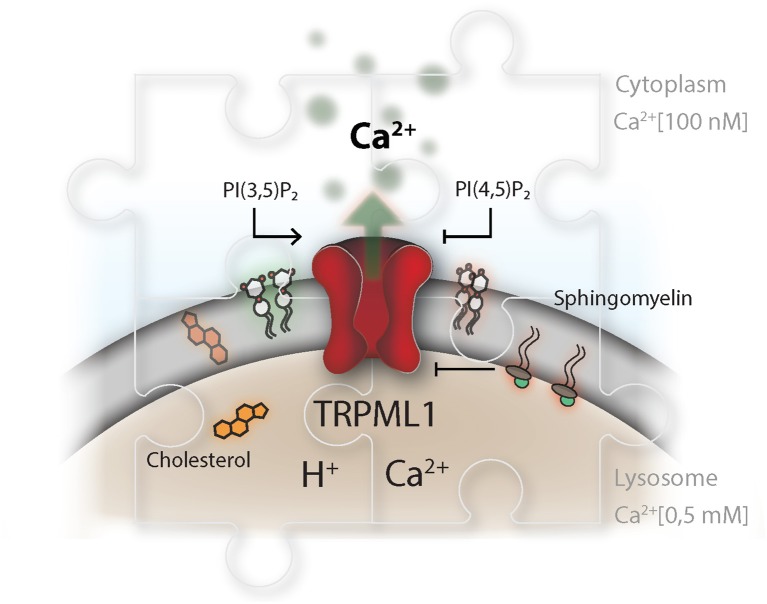
TRP channels as master regulators in the endolysosomal compartment. The TRP channel conductance is affected by a broad range of stimuli and integrates information about pH and signals from within the lipid bilayer, as well as the surrounding cytosol and vesicular lumen. This is illustrated for TRPML1, which is described to be activated by phosphatidylinositol-(3,5)-bisphosphate, PI(3,5)P_2_, and inhibited by phosphatidylinositol-(4,5)-bisphosphate. PI(3,5)P_2_ and PI(4,5)P_2_ play an delicate role in controlling lysosomal membrane dynamics, hence their synthesis needs to be highly coordinated in time and space. Disparity in the concentration of TRP ligands, in the context of lysosomal storage disorders, impairs proper lysosomal lipid transfer, trafficking and calcium signaling capacity. Niemann-Pieck disease type A/B is characterized by lysosomal accumulation of sphingomyelins (SM). In this disease, SM has been suggested to be the causing agent for the grave pathogenic conditions due to its presumable antagonistic function on TRPML1. On the other site, Niemann Pieck type C is denoted by a defect in cholesterol transporters and might also affect SM levels in the lysosome.

TRPML1 is a non-selective calcium-permeable TRP cation channel resident in the lysosomal compartment. Mutations in the MCOLN1 gene, which encodes TRPML1, lead to Mucolipidosis type IV which is a lysosomal storage disorder. Affected patients present with severe neurological and ophthalmological symptoms (129). On a molecular level, loss of mucolipin-1 activity impairs lipid efflux from the lysosomal compartment and results in abnormal endolysosomal transport (130). Phosphatidylinositol-(3,5)-bisphosphate (PI(3,5)P_2_) has been identified as an endogenous agonist of the mucolipin TRP channel subfamily and can trigger lysosomal calcium efflux (131). PI(3,5)P_2_ is produced by a phosphatidylinositol-(3)P 5-kinase termed PIKfyve (132). The role of PIKfyve, upstream of TRPML1, as a regulator of lysosomal positioning and fusion has been well-established (70, 133, 134). On the other hand, PI(4,5)P_2_, which can be found as transient pools on lysosomes and autophagosomes (87, 135), and sphingomyelin have antagonistic properties on TRPML1 activity (136, 137) (Figure 3).

As demonstrated in cell-free assays, luminal calcium is a prerequisite for proper formation of dense-core lysosomes from the endolysosomal compartment (80). Potentially, activation of TRPML1 by PI(3,5)P_2_, together with low lysosomal pH, may trigger luminal calcium efflux and recruitment of calmodulin and other hitherto unidentified protein machineries to the surface of the endolysosome, which then may facilitate membrane tubulation or scission between the parental organelle and newly formed protolysosome (67, 138, 139). Thus, TRPML1 may directly alter the luminal calcium concentration and influence lysosomal size and re-formation (88, 134). Activation of TRPML1 requires a close spatiotemporal adjustment of agonistic PI(3,5)P_2_, antagonistic PI(4,5)P_2_ and sphingomyelin concentrations in the lysosomal membrane. Therefore, lysosomal fission and fusion events are precisely coordinated by lysosomal membrane lipid concentrations.

TRPML1-dependent lysosomal calcium efflux is also involved in the activation of the transcription factor TFEB. Originally, TFEB was known to be a mTOR-dependent master regulator of a gene network (CLEAR network), associated with lysosomal biogenesis and autophagy (69, 140). The classical role of the microphthalmia/transcription factor E MiT/TFE- family of transcription factors has been extended, since the two members, TFEB and TFE3, have been linked as transcriptional regulators of innate and adaptive immune responses (73, 141). In macrophages, pathogen challenge and engagement of Fcγ- or toll like receptors initiates TFEB and TFE3-dependent expression of lysosomal genes, autophagy-related genes and pro-inflammatory effector molecules, such as CCL5, TNFα and IL-1β or IL-6 (142–145). Interestingly, MCOLN1, the gene coding for TRPML1 is under the transcriptional control of TFEB itself (140). Thus, in regard to immune cell activation, the TRPML1-TFEB axis might also have important implication for the transcriptional regulation of NK cell effector functions. Acute activation of NK cells could trigger TRPML1-mediated lysosomal calcium flux. Subsequently, these calcium signals can lead to nuclear translocation of TFEB/TFE3, which initiates TRPML1 upregulation and secretory lysosomal biogenesis and reformation, as well as transcription of additional pro-inflammatory molecules, similar to what has been demonstrated in macrophages. In NK cells, the exact pathways and regulatory networks of the MiT/TFE transcription factors have yet to be dissected.

Since TRPML1 is such a central player in the regulation of lysosomal biogenesis and secretory lysosomal function, it is not surprising that this channel protein might have important implications for the immune system, as already demonstrated in NK cells and murine macrophages (25, 88). Based on RNA sequencing data, TRPML1 is expressed at relatively constant levels across mature and immature NK cell subsets (25). Our laboratory recently demonstrated that the highly functional educated NK cells (express inhibitory KIR for self HLA class I) have larger dense-core lysosomes, which converge closer to the MTOC and accumulate more lysosomal calcium and granzyme B, as compared to NK cells that lack inhibitory receptors to self HLA and therefore receive tonic activation input. The phenotype of educated NK cells could be mimicked by pharmacological as well as genetic interference of the PIKfyve-TRPML1 axis and the lysosomal calcium content. These results point to a role for lysosomal calcium and the TRPML1 calcium channel in the remodeling of dense-core lysosomes.

### TRPML2

TRPML2 is the second member of the Mucolipin subfamily of TRP channels. As all members of the mucolipin subfamily TRPML2 also exhibits a pore domain and is permeable to sodium and calcium. TRPML2 is encoded by the MCOLN2 gene, the expression pattern of which seems to be more restricted to the lymphoid and myeloid tissue. More specifically this selected set of tissues includes thymus, spleen and immune cells. From all three members of the mucolipin subfamily, TRPML2 shows the most limited tissue distribution, compared to the other members of the subfamily (146). Based on electrophysiological data, TRPML2 activity is reduced at low luminal pH and reaches its maximum at neutral luminal pH (24). TRPML2 can be activated as previously introduced for TRPML1, by the phosphatidylinositol-(3,5)-phosphate PI(3,5)P2 (131, 147). On the other hand in structural studies on TRPML2 it was claimed that the pre-pore regions of TRPML1 and TRPML2 are structurally more similar to each other than to TRPML3 under acidic conditions (148). While TRPML1 activity reaches is maximum at highly acidic luminal pH, TRPML3 maximal activity is found at neutral pH. Both TRPML2 and 3, in contrast to TRPML1 reside in early and recycling endosomal compartments hence would be exposed to less acidic conditions. In conclusion, the early endosomal and recycling endosomes provide a neutral pH, which promotes TRPML2 activity (24).

The selective expression of MCOLN2 in lymphatic and myeloid cells suggests that this calcium channel is involved in regulating immune cell function (149). TRPML2 resides in Arf6^+^ recycling endosomes and is associated with recycling of plasma membrane proteins (150). In murine macrophages TRPML2 expression is connected to activation status and modulates the recruitment and chemokine secretion in response to pro-inflammatory stimuli. The latter effect has been shown by knock-out studies and upon specific pharmacological intervention (24, 151). There are many outstanding questions regarding the role of the intracellular TRPML2 localization and its impact on TRPML1 activity and effector functions in general. TRPML2 is linked to the recycling endosomes and observations support a role in the regulation of innate immune cell function (149). In NK cells, MCOLN2 expression reaches its peak in the terminally differentiated adaptive CD57^+^ NKG2C^+^ NK cell subset but is also detected in various NK cell lines (152, 153). It is tempting to speculate that TRPML2 modulates chemokine secretion in activated NK cells and perhaps to a greater extent in adaptive NK cells.

### TRPM2

In addition to the mucolipins, the transient receptor potential melastatin 2 (TRPM2) channel can be detected in a wide range of immune cells and has implications for granzyme B secretion and antitumor activity of NK cells among others (21, 111, 118). TRPM2 is a calcium-permeable cation channel and responsive to reactive oxygen species (ROS) and the second messenger ADP-ribose (ADPR) (154, 155). Furthermore, the channel can be synergistically gated by a family of second messengers, derived from the ectoenzyme CD38, which comprises: cyclic ADP-ribose (cADPR), nicotinic acid adenine dinucleotide phosphate (NAADP) and ADPR (154–157). TRPM2 is resident in the plasma membrane and can also be found on the lysosomal membrane (157, 158). Several studies outlined the proinflammatory character of TRPM2 and its importance for innate and adaptive immune cells (118, 120, 156). A study examined the effects of ADPR on TRPM2 modulation in murine NK cells and found that TRPM2 knock-out mice exhibit a defect in their antitumor responses (21). The cytotoxic activity of NK cells strongly correlates with their ability to secrete granzyme B and perforin-containing granules. Upon tumor encounter, CD38 and TRPM2 localizes to secretory lysosomes in mouse NK cells (21, 159). Mechanistically, CD38-derived ADPR has been associated with sustained TRPM2-derived calcium mobilization, which is a prerequisite for granule polarization and degranulation (21). Intervention of ADPR production by ADPR antagonists or genetic manipulation have been associated with lower intracellular calcium mobilization and reduced effector responses in mouse NK and T cells (21, 119). Similar data was obtained in T cells, where NAADP was associated with granule polarization and directed degranulation (26). The studies on TRPM2 showed a direct link of how a cation channel can modulate crucial processes like antitumor responses (21, 119).

Collectively, the studies discussed above highlight a potentially important role for lysosomal calcium channels, particularly TRP channels, in the formation and remodeling of secretory lysosomes and in tuning various effector functions in immune cells. However, more data from cells of human origin are needed to draw conclusions about the pharmacological benefit and potential of TRP agonists and antagonists in humans. The lack of specific antibodies and low expression levels of most TRP channels make the work with TRP channels challenging. Therefore, as we shall discuss in the next section, new tools are needed to accelerate the accumulation of knowledge in the field (160).

## Methodological Advances For Assessing The Function of TRP Channels in Immune Cells

The tight connection between the endolysosomal system and the pathogenesis of metabolic, neurological and infectious human diseases has motivated increasing efforts to advance the tool-box for studies of lysosomal biology. Over 70 lysosomal storage diseases (LSD) have been described and linked to mutations in lysosomal proteins, highlighting the urgency to decipher the lysosomal function more in depth (161–163). Overall, the lack of knowledge of the precise function of malfunctional lysosomal gene products results in poorly available treatment options (163). Early experiments mapping the proteome of isolated secretory lysosomes from NK cells generated valuable information about granule specific proteins (164). Similar but more targeted approaches were applied to make the endolysosomal membrane composition more accessible and revealed the identity of more than 70 lysosomal ion channels and transporter proteins (165, 166).

Typically, electrophysiological properties, such as the ion selectivity of channel proteins are studied by patch-clamping, but this has remained challenging for intracellular, endolysosomal proteins. The combination of genetic manipulation, or advancement thereof with small molecules, which selectively enlarge endolysosomal organelles, together with patch-clamp technology enabled interrogation of the properties of such intracellular ion channels in great detail also in immune cells (167–169). In particular, the research on the mucolipin family of TRP cation channels gained momentum by the development of selective pharmacological agonists. These small molecules can also assist in validating established patch-clamp approaches or function as lead structures for potential drug candidates, respectively (22, 170, 171). The development of a series of genetically-encoded calcium indicators (GECI), such as the FRET-based Cameleon-type (172) or the single GFP-based GCaMP-family (173, 174), fused to specific TRP channels, hold promise to delineate calcium signals in immune cells with subcellular resolution in time. The functional principle of the GCaMP reporters is based on circulary permutated EFGP (cpEGFP), N-terminally fused to the M13 peptide obtained from myosin light chain kinase and C-terminally connected to calmodulin (CaM) (175). Initially, the reporter exhibits weak fluorescent activity, but upon calcium binding to the CaM domain, CaM binds to the M13-peptide and the cpEGFP undergoes a conformational change, which induces a drastic increase in fluorescent activity (173, 175, 176). The advantage of these sensors lies in their stable expression levels and the possibility to target them to subcellular organelles like lysosomes (69, 136). Modifications of the GFP-based GECIs, can also be used as organellar-entrapped sensors, to survey luminal calcium concentrations (177). Many of these calcium reporters have a single wavelength and can be combined with further reporters like genetically-encoded pH sensors (178). Further novel technologies, like the usage of induced pluripotent stem cell (iPSC)-derived NK cells can help to circumvent the challenging gene-editing of primary NK cells. iPSCs can more easily be genetically manipulated and function as a human model system for studying TRP channels in NK cells (179, 180). Altogether, these advances hold promise to generate ground-breaking discoveries concerning the spatiotemporal contribution of TRP channels to the dynamic modulation of immune cell function.

## Concluding Remarks

Recent experimental data has challenged our current understanding of lysosomes. Until recently, these organelles have been seen as a static terminal storage unit of the endosomal pathway, providing degradative capacity to the cell. In cytotoxic lymphocytes, lysosomes act also as secretory lysosomes, which can be released upon target cell encounter. The lysosomal homeostasis and biogenesis are complex and calcium signaling plays a critical role. In NK cells the lysosomal compartment undergoes constant remodeling that is under influence by surface receptors binding to self MHC molecules. Specifically, inhibitory signaling through KIR and CD94/NKG2A during NK cell education is tightly associated with retention of lysosomal matrix proteins and accumulation of perforin and granzyme B in a unique pool of dense-core secretory lysosomes. The consequence is an enhanced lysosomal signaling capacity and increased NK cell effector responses. The role of TRP channel members as regulators of lysosomal calcium fluxes and the subsequent effects on lysosomal fission and fusion events are gaining increasing attention. New tools, including highly specific small molecule TRP agonists and antagonists, as well as organelle-specific, genetically-encoded calcium sensors hold promise to advance the field.

Biological insights into the dynamic regulation of lysosomal signaling may pave the way for new means to boost function of cytotoxic lymphocytes in cancer immunotherapy. In this context, TRP channels represent promising targets for manipulation by means of pharmacological or genetic perturbation (25). The mucolipin subfamily of TRP cation channels could potentially be targeted to modify the rate of lysosomal biogenesis or, as described for TRPML2 in macrophages, to modulate the secretion of chemokines in immune effector cells (24, 151). It may also be possible to build functional potential in lymphocytes through manipulation of the lysosomal matrix, which influence loading of positively charged effector molecules such as granzyme B as well as calcium (181, 182). Another attractive possibility is to indirectly affect lysosomal signaling capacity through tuning of NK cell education by agonistic stimulation of inhibitory or activating receptors on NK cells. A key challenge for all these efforts is to determine the right kinetics since long term silencing/inhibition of calcium channels may be detrimental to the cell. Intermittent, pulsatile interference may be superior to chronic engagement given the rapid calibration of immune cell reactivity to external input (183). Despite these challenges, the insight that TRP channels contribute to remodeling of the interior of immune effector cells hold promise for the discovery of new therapeutic targets in cancer immunotherapy.

## Author Contributions

DC wrote the manuscript and made the illustrations. All authors contributed to the conceptual framework of the review and to the writing of the manuscript.

## Conflict of Interest

JG is an employee at Fate Therapeutics Inc. (San Diego, USA). K-JM is a consultant and advisor at Fate Therapeutics. The remaining authors declare that the research was conducted in the absence of any commercial or financial relationships that could be construed as a potential conflict of interest.
